# Common Pollen Modulate Immune Responses against Viral-Like Challenges in Airway Coculture Model

**DOI:** 10.1155/2023/6639092

**Published:** 2023-11-06

**Authors:** Tarleena Tossavainen, Maria-Viola Martikainen, Hanna Loukola, Marjut Roponen

**Affiliations:** Department of Environmental and Biological Sciences, University of Eastern Finland, Kuopio, Finland

## Abstract

Recent research indicates that exposure to pollen increases the risk and severity of respiratory infections, while studies also suggest that it may possess a protective function. Our aim was to investigate how exposure to common pollen modifies airway cells' responses to viral- or bacterial-like challenges and vice versa. Cocultured A549 and THP-1 cells were exposed to three doses of four different pollens (*Alnus glutinosa*, *Betula pendula*, *Phleum pratense*, or *Ambrosia artemisiifolia*) and subsequently to Toll-like receptor (TLR) ligands mimicking bacterial and viral challenges (TLR3, TLR4, TLR7/8). The stimulation experiment was replicated in reverse order. Toxicological and immunological end points were analyzed. When cells were primed with pollen, especially with grass (*P. pratense*) or weed (*A. artemisiifolia*), the ability of cells to secrete cytokines in response to bacterial- and viral-like exposure was decreased. In contrast, cells primed with viral ligand TLR7/8 showed greater cytokine responses against pollen than cells exposed to ligands or pollen alone. Our results suggest that pollen exposure potentially weakens immune reactions to bacterial- or viral-like challenges by modulating cytokine production. They also indicate that TLR7/8-mediated viral challenges could elicit exaggerated immune responses against pollen. Both mechanisms could contribute to the acceleration and complication of infections during the pollen season.

## 1. Introduction

With each passing year, climate change poses a growing threat to our respiratory health, particularly during the pollen season. For individuals who suffer from allergies, exposure to respiratory viruses during this time can be especially challenging. Climate change increases exposure to pollen [1] and pathogens [2, 3], leading to even more prevalent exposure to both risk factors. The potential synergistic effects of these factors on health could result in an increase and exacerbation of various respiratory problems.

Recent research has shed light on the complex interactions between pollen and viruses, highlighting the need for further investigation, as reviewed in a study by Martikainen et al. [4]. One study found that exposure to high pollen concentrations explained more than 40% of the increase in severe acute respiratory syndrome coronavirus 2 (SARS-CoV-2) infection rate in synergy with humidity and temperature [5]. On the other hand, an inverse relationship between pollen levels and the incidence of influenza-like illnesses has been observed [6–8]. It is still unclear how pollen exposure contributes to the viral infectiveness or severity of diseases. Few *in vivo* and *in vitro* studies have assessed how pollen interferes with antiviral immunity [9–11]. More research is needed, however, to explain the immunological and toxicological mechanisms behind the effects of simultaneous exposure to pollen and viruses. Furthermore, the differences between pollen species and the importance of the timing of the exposure have not been comprehensively studied. Environmental factors, such as higher temperature and higher solar radiation combined with hay fever incidence, seem to limit the spreading of COVID-19 [12], indicating that allergic diseases without pollen exposure may reduce susceptibility to viral infection. This is supported by observation that allergic diseases are associated with lower rates of COVID-19 hospitalizations [13].

To increase our understanding of the interactions between pollen and pathogenic exposures, we conducted an *in vitro* study investigating how exposure to different types of pollen affects airway cells' ability to respond to secondary exposure to bacterial- and viral-like challenges. To replicate the first line of defense of lungs in an *in vitro* model, we used a submerged coculture of alveolar epithelial cells (A549) and macrophages (THP-1). To mimic bacterial and viral stimuli, we used TLR3, 4, and 7/8 ligands. Furthermore, our objective was to determine whether priming with viral or bacterial ligands affects cells' responsiveness to subsequent exposure to pollen. Therefore, the exposure experiments were replicated in reverse order. Our results provide new insight into effects of coexposure on the airway integrity and immunity, paving the way for further studies aiming to improve our understanding of managing respiratory health during pollen season.

## 2. Methods

### 2.1. Preparation of Pollen Extracts and Ligands

Pollen from four different species were studied: two trees, black alder (*Alnus glutinosa)* and white birch (*Betula pendula)*; a grass, timothy (*Phleum pratense*); and a weed, common ragweed (*Ambrosia artemisiifolia*) (all purchased from Allergon, Angelholm, Sweden). Pollen was suspended in Dulbecco's Modified Eagle Medium (DMEM) (Sigma-Aldrich) at the concentration of 100 mg/ml and incubated for 30 min at 37°C with vortexing every 10 min. After incubation, pollen suspension was centrifuged (10 min, 6,300 × *g*, RT) and filtered (0.22 *µ*m filter, Biofil) similarly in a study by Gilles et al. [14]. Supernatants were stored at −20°C.

We used polyinosinic-polycytidylic acid (Poly (I:C), Miltenyi Biotec) to mimic virus-derived double-stranded RNA (TLR3 agonist), lipopolysaccharide (LPS) (Sigma-Aldrich) to mimic Gram-positive bacteria (TLR4 agonist), and synthetic single-stranded RNA oligoribonucleotide (ORN R-0006, Miltenyi Biotec) to mimic single-stranded viral RNA (TLR7/8 agonist). These agonists are often used in research to study immune system activation, inflammation, and host–pathogen interactions, as they mimic the molecular patterns present in pathogens and activate Toll-like receptors (TLRs). All ligands were prepared according to the manufacturers' instructions.

### 2.2. Coculture Airway Cell Model

Human alveolar epithelial cell line A549 (ATCC® CCL-185™, Germany) and THP-1 human monocytes cells (DSMZ ACC 16, Germany) were maintained in DMEM, supplemented with 10% fetal bovine serum (FBS), 2 mM L-glutamine, and 100 U/ml penicillin/streptomycin (all Sigma-Aldrich, USA). For cocultures, monocyte-like THP-1 cells were differentiated into macrophage-like cells with phorbol 12-myristate 13-acetate (PMA) (Sigma-Aldrich) before the experiments, as described in Rönkkö et al. [15].

For the experiments, A549 cells were seeded to the 12-well plates in 1 ml of medium (120,000 cells/ml). After cell attachment period of 4 hr, the media were removed and differentiated THP-1 cells (24,000 cells/ml/well) were seeded on top of A549 cells and allowed to attach. Cocultured cells were then incubated for approximately 45 hr before exposure. The seeding densities of A549/THP-1 coculture were selected to achieve a cell density of around 400,000–600,000 cells/ml at the end of the 24-hr exposure period, as in a study by Kasurinen et al. [16]. Medium was replaced 1 hr before the first exposure.

### 2.3. Pollen and Ligand Exposures

**Figure 1:**
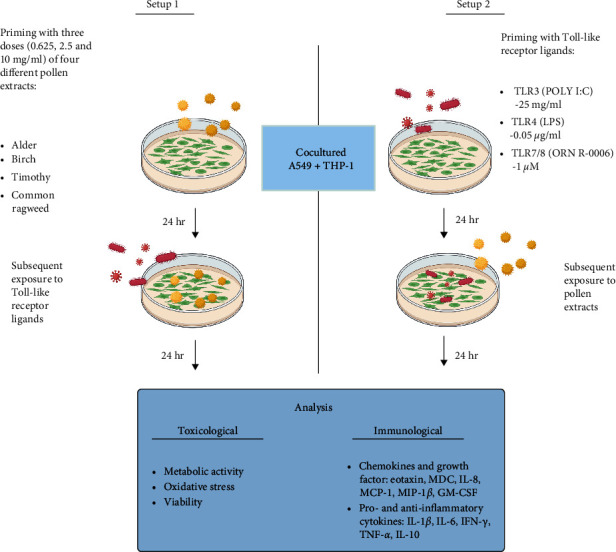
Two exposure setups. In setup 1, cocultured A549 and differentiated THP-1 cells were first exposed to three doses of pollen extracts (final concentrations: 0.625, 2.5, and 10 mg/ml) for 24 hr followed by the secondary exposure to bacterial and viral ligands (Poly (I:C) 25 *µ*g/ml, LPS 0.05 *µ*g/ml, and ORN R-0006 1 *µ*M) for 24 hr. In setup 2, exposures were performed in an opposite order. After exposure, toxicological and immunological end points were measured. Figure created with https://BioRender.com.

Experiments were performed in submerged culture and two exposure setups were used (Figure 1). In setup 1, cells were first exposed to three doses of pollen extracts (final concentrations: 0.625, 2.5, and 10 mg/ml) for 24 hr (5% CO_2_, 37°C), followed by the secondary exposure to bacterial and viral ligands (LPS 0.05 *µ*g/ml, Poly (I:C) 25 *µ*g/ml, and ORN R-0006 1 *µ*M) for 24 hr (setup 1, Figure 1). The pollen doses were chosen to be in line with other *in vitro* studies investigating the effects of pollen on airway cells [9, 17]. Before this study, the selected doses were further tested to ensure that they are nontoxic in the cell culture conditions applied in this study. Doses of TLR agonists were the same as used by Shahbaz et al. [18]. Medium was not changed between exposures. In the second setup, exposures were performed in an opposite order (setup 2, Figure 1). For the readability of the manuscript, we will refer to these (priming-subsequent exposure) exposure systems as coexposures in the Results and Discussion sections. All experiments were performed at least three times with two replicates per exposure.

### 2.4. Membrane Permeability, Oxidative Stress, and Cellular Metabolic Activity

After exposure, cells were detached by trypsinization [15] and analyzed for cell viability, metabolic activity, and production of reactive oxygen species (ROS). Cell viability was analyzed as membrane permeability using propidium iodide (PI), metabolic activity was measured using MTT assay, and the levels of intracellular ROS were measured using 2′, 7′-dichlorofluorescein (DCF) assay as described by Kasurinen et al. [16]. PI fluorescence (540 nm excitation and 610 nm emission), DCF fluorescence (485 nm excitation and 530 nm emission), and MTT absorbance at 570 nm were measured with Synergy H1™ reader (USA). Metabolic activity and oxidative stress responses are expressed as percentages of fluorescence compared to the control. Cell membrane permeability is expressed as a percentage of intact cells compared to the control. All analyses were run in duplicate.

### 2.5. Cytokine Analysis

Cell culture medium was collected and stored at −80°C for cytokine analyses. Levels of eotaxin, MCP-1, MDC, MIP-1*β*, interferon-*γ* (IFN-*γ*), interleukin-1*β* (IL-1*β*), IL-6, IL-10, tumor necrosis factor-*α* (TNF-*α*), and granulocyte-macrophage colony-stimulating factor (GM-CSF) in media were measured using MSD Proinflammatory Panel 1 (human) U-PLEX Kit (MSD, Rockville, MD, USA) according to manufacturer's instructions, using reagents provided with the kit. Plates were analyzed with Meso Scale Discovery Sector Imager™ 2400A with Discovery Workbench® 3.0.18 software. In addition to the beforementioned cytokines, the secretion of IL-8 was determined by enzyme-linked immunosorbent assay (Thermo Fisher Scientific) according to the manufacturer's instructions and measured with Synergy H1™ reader.

The detection limit (DL) was defined for each cytokine separately. Distributions of cytokines and detection ranges are shown in Tables S1 and S2. Samples with concentrations below the DL but over zero were given value corresponding to the DL of the respective cytokine assay. Sixty-five percent of stimulated IL-6, 79% of stimulated MCP-1, and 54% of MIP-1*β* were above the upper DL; these three cytokines were excluded. Data from cytokine measurements are expressed as fold changes (changes in the cytokine production between unexposed and exposed cells).

### 2.6. Statistical Analysis

The data have been presented as means ± standard error of means (SEM). All pairwise comparisons were done with the Mann–Whitney *U* test. Statistical analyses were performed using SPSS Statistics version 27 software (IBM Corporation, USA). Values of *P* < 0.05 were considered as statistically significant.

## 3. Results

### 3.1. Pollen Exposure Modifies Intracellular Oxidative Responses against Pathogen-Like Exposures (Setup 1)

To test whether studied pollen extracts affect the function of the airway barrier and how it responds to pathogen-like exposures, cocultures were first incubated with pollen extracts and subsequently exposed to TLR ligands (setup 1).

**Figure 2:**
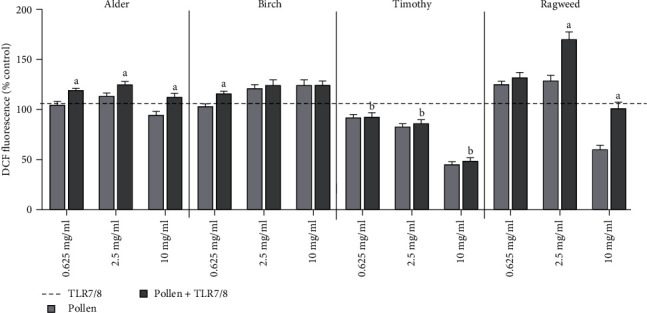
Oxidative stress responses in setup 1. Levels of reactive oxygen species after priming with different pollen extracts and subsequent TLR7/8 exposure (setup 1, *N* ≥ 3) were measured using DCF assay. Figure shows mean ± SEM. Significance was assumed at *p* < 0.05. a indicates significant difference from pollen, and b indicates significant difference from TLR7/8.

Intracellular ROS production was decreased in cells exposed to timothy pollen alone, and subsequent exposure to TLR7/8 was not able to restore oxidative responses (Figure 2). The same effects were observed after subsequent exposure to TLR3 and TLR4 (Table S3).

Metabolic activity decreased in response to timothy and ragweed at the highest dose, but no further decrease was observed after subsequent exposure to ligands (Table S3). Alder, birch, timothy, or coexposures to these pollen extracts and ligands did not affect cell viability (Table S3). Instead, the highest dose of ragweed decreased the percentage of living cells.

### 3.2. Priming with Pollen Decreases the Release of Immunological Mediators in Response to Pathogen-Like Exposure (Setup 1)

**Figure 3:**
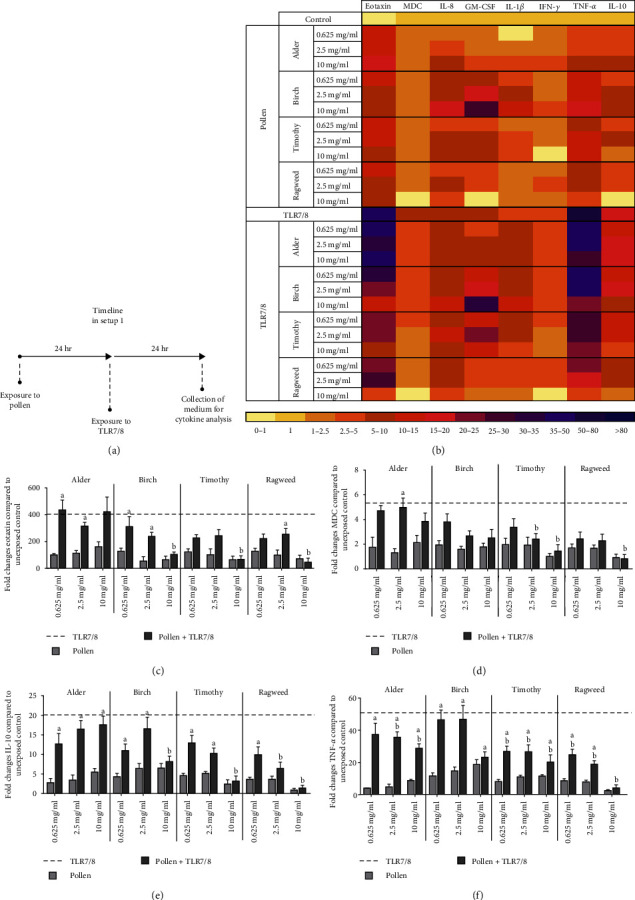
Cytokine production in setup 1. (a) Cocultured cells were first exposed to three doses (0.625, 2.5, 10 mg/ml) of four different pollen extracts (24 hr) and subsequently to Toll-like receptor ligand 7/8 (24 hr) (setup 1, *N* ≥ 3). (b) Heat map depicting the fold changes of eotaxin, MDC, IL-8, GM-CSF, IL-1*β*, IFN-*γ*, TNF-*α*, and IL-10. Eotaxin values were divided by 10 to scale them for the heat map range. (c)–(f)) Detailed results of eotaxin, MDC, IL-10, and TNF-*α*. Figures show mean ± SEM. a indicates significant difference from pollen, and b indicates significant difference from TLR7/8.

To study the effect of pollen extracts on immunological responses against pathogen-like exposures (setup 1), we measured several chemokines, growth factors, and pro- and anti-inflammatory cytokines (Figure 3(b)–3(f)). Of the studied pollen, birch induced the highest secretion of several cytokines.

Priming the cells with the pollen extract, especially with timothy or ragweed, decreased the ability of cells to secrete chemokines in response to pathogen-like exposure, that is, coexposed cells produced less eotaxin and MDC than those exposed to TLR7/8 alone (Figure 3(b)–3(d)). Similar, albeit not as strong, effects were seen in cells coexposed to pollen and TLR4 (Figure S1).

Coexposure to pollen extracts and TLR7/8 also decreased the secretion of proinflammatory IFN-*γ* (Figure 3(b)) and TNF-*α* (Figures 3(b) and 3(f)). A comparable phenomenon was seen in IL-1*β* albeit only in cells primed with ragweed.

Cells primed with pollen, especially timothy or ragweed, were not capable of secreting anti-inflammatory IL-10 in response to TLR7/8 or TLR4 when compared to ligands alone (Figures 3(b) and 3(e), Figure S2). TLR3 induced only a modest increase in cytokine production, and the release of cytokines was comparable between coexposed cells and cells exposed to pollen extracts alone (Figures S3 and S4).

### 3.3. TLR4 and TLR7/8 Priming Affects Cells' Ability to Produce Reactive Oxygen Species (Setup 2)

We also wanted to determine whether priming with viral or bacterial ligands affects cells' ability to respond to subsequent exposure to pollen (setup 2).

**Figure 4:**
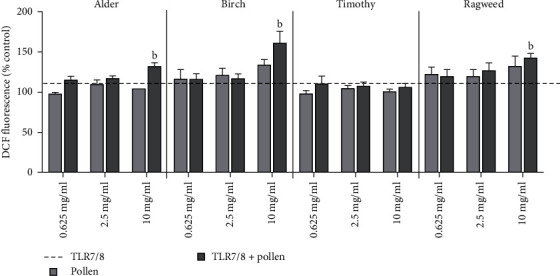
Oxidative stress responses in setup 2. Levels of reactive oxygen species after priming with TLR7/8 and subsequent pollen exposure (setup 2, *N* ≥ 3) were measured using DCF assay. Figure shows mean ± SEM. Significance was assumed at *p* < 0.05. a indicates significant difference from pollen, and b indicates significant difference from TLR7/8.

Coexposure to TLR7/8 and alder, birch, or ragweed pollen extracts increased the production of ROS compared to TLR7/8 alone (Figure 4). Similar effects were seen in TLR4-primed cells following birch and ragweed pollen exposure (Table S4). Priming with ligands did not significantly alter oxidative responses induced by pollen. Ligands, pollen, or coexposure with them did not affect cell viability, and only a few changes in metabolic activity were observed (Table S4).

### 3.4. Priming with Viral Ligand TLR7/8 Enhances Cytokine Production after Subsequent Exposure to Pollen (Setup 2)

**Figure 5:**
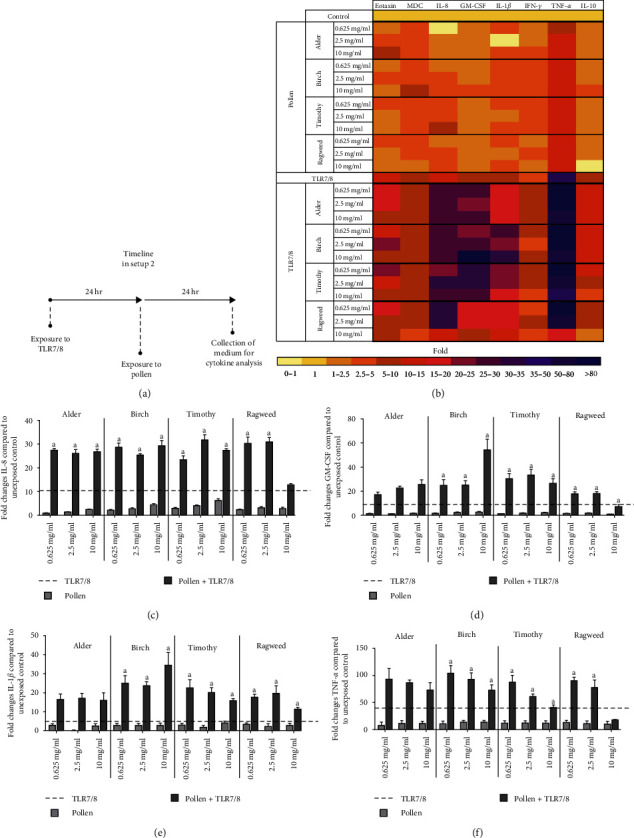
Cytokine production in setup 2. (a) Cocultured cells were first exposed to Toll-like receptor ligand 7/8 (24 hr) and subsequently to three doses (0.625, 2.5, 10 mg/ml) of four different pollen extracts (24 hr) (setup 2, *N* ≥ 3). (b) Heat map depicting the fold changes of eotaxin, MDC, IL-8, GM-CSF, IL-1*β*, IFN-*γ*, TNF-*α*, and IL-10. (c)–(f) Detailed results of IL-8, GM-CSF, IL-1*β*, and TNF-*α*. Figures show mean ± SEM. a indicates significance from pollen, and b indicates significance from TLR7/8.

We also measured cytokine levels to further determine whether pathogen-like exposures affect cells' ability to elucidate immunological responses against pollen. Exposure to TLR7/8 ligand alone induced significant cytokine responses in cells, while cytokine levels remained low following exposure to pollen extracts alone (Figure 5(b)–5(f)). Interestingly, all studied pollen triggered a massive release of IL-8, GM-CSF, IL-1*β*, TNF-*α*, and IL-10 in cells primed with TLR7/8 but not with TLR3 or TLR4 (Figures S5–S8). Inverse dose responses in the levels of several cytokines were observed in cells coexposed to TLR7/8 and ragweed or timothy.

## 4. Discussion

While there have been numerous investigations of the consequences of pollen and respiratory pathogens on lung physiology separately, the comprehensive understanding of their synergistic effects on airway function and integrity has been relatively understudied. To our knowledge, this study is the first one assessing both aspects of the phenomenon in airway cell model: we examined how pollen exposure affects the reactions of cocultured A549 and THP-1 cells to viral or bacterial challenges and, on the other hand, how exposure to viral ligands modulates responses to pollen. We show that common pollen weaken the capability of cells to respond to exposure to viral ligands by decreasing the production of ROS and cytokines. Conversely, prior exposure to viral ligands leads to an unnecessarily strong immune response by enhancing cytokine production against pollen.

### 4.1. Interactions between Pollen and Pathogens Modulate Cellular Responses

Our findings highlight the importance of the synergistic effects of pollen and pathogens and, more specifically, the order in which airways encounter these challenges. Priming cells with pollen impaired the secretion of multiple cytokines following pathogen-like exposures. Interestingly, this phenomenon was seen in all types of cytokines, i.e., chemokines, pro- and anti-inflammatory cytokines. Also, a study by Gilles et al. [10] showed that simultaneous exposure to pollen and viral-like stimulation decreased chemokine responses. Even though a causal connection between infection rate and pollen has not yet been fully established and the mechanisms are not clear, pollen exposure seems to suppress antiviral immunity *in vitro* and *vivo*. In a recent study by Fneish et al. [11], birch pollen treatment enhanced human cytomegalovirus (HCMV) infection in monocyte-derived dendritic cells but did not affect antiviral cytokine IFN-*α* responses. Pollen-derived proteases have also been shown to cause irreversible damage to the equine respiratory epithelium, which can benefit viruses and lead to an increase in infections [9]. Our findings and previous studies point to the possibility that pollen may contribute to the infectivity of pathogens.

To our knowledge, this study is the first to report how airway cells primed with pathogen-like ligands react to pollen exposure. We showed that the production of cytokines, especially proinflammatory TNF-*α* and IL-1*β*, was amplified in cells that were primed with viral ligand and subsequently exposed to pollen. This response resembles a cytokine storm linked with respiratory infections. Similar results have been reported earlier using other cell lines. For example, Fneish et al. [11] found that cotreatment with HCMV and birch pollen increased antiviral IFN-*α* protein production compared to viral exposure alone in dendritic cells. Pollen has also been shown to induce the reactivation of a latent gammaherpesvirus in murine macrophages [19]. Interestingly, Wisgrill et al. [20] recognized an exaggerated antiviral response to Bet v 1 stimulation in peripheral blood mononuclear cells (PBMCs) of birch-allergic patients. Overall, an overactivated antiviral response during the pollen season may lead to robust inflammatory reactions that could affect the phenotype of the disease. The importance of these findings in relation to sensitization and the development of allergic diseases remains to be disentangled.

Several pollen has NADPH oxidase activity and can induce the production of ROS in airway epithelium, thus inducing allergic inflammation [21–23]. Dendritic cells exposed to ragweed also produce ROS [24]. The effects of coexposure with viral-like pathogens and pollen on ROS production or metabolic activity of the cells have not been assessed earlier. We showed that exposure to timothy pollen decreased intracellular oxidative responses to pathogen-like exposures. We also observed that the metabolic activity of cells decreased when cells were primed with timothy or ragweed pollen. The changes in metabolic activity and ROS production appeared to be induced by pollen, and they could not be explained by the reduction in cell viability.

Altogether, previous studies and our results suggest that pollen exposure may be able to compromise defense mechanisms against pathogens, potentially leading to a higher probability of infections and more severe symptoms during the pollen season. Results of population-based studies indicate, however, that pollen exposure can decrease the risk of COVID-19 infection [25]. An inverse relationship between the presence of allergic diseases and the rate of COVID-19 hospitalization has also been observed [13, 26]. Therefore, there is a need for further investigation into the interactions between different viral pathogens, pollen species, and the allergic status of individuals, as well as the underlying immunological mechanisms.

Asthma and allergies are linked to the reduced expression of the angiotensin–converting enzyme 2 (ACE2) in the airways, the best known entry point for SARS-CoV-2 [25, 27]. This provides one potential mechanism by which allergies may decrease susceptibility to severe SARS-CoV-2 infection. Unfortunately, our cell model was not optimal for studying the expression of the ACE2 receptor. Moving forward, it would be interesting to investigate how simultaneous exposure to pollen and pathogens affects the expression of this receptor. It is noteworthy that the cells we used represent secondary cell lines and cannot be utilized when studying the effects of different health statuses on immune outcomes. Novel 3D primary airway cell models could, however, be used to assess whether interactions between pollen and pathogens differ in tissues from allergic and nonallergic donors and to explore the mechanisms that could explain protection against severe infections.

### 4.2. Pollen Species Differ in Respect to Their Antiviral-Like Properties

We used four common pollen that trigger allergies (alder, birch, timothy, and common ragweed) in our study. These species were also chosen since they cover the majority of pollination season. Alder is one of the first pollinators in the spring, while birch, which is one of the most common allergenic trees in the Northern Hemisphere, including Europe and North America, follows alder. Grasses, especially timothy, are also common causes of hay fever in Europe. Last, we chose common ragweed as it is one of the most allergenic plants. While it is a native species of North America and a major cause of hay fever there, it has also spread to Southern and Southeastern Europe. Climate change has recently raised concerns about the increased distribution of ragweed species to new areas. In our study, we noticed that pollen species differ regarding their toxicological and immunological properties. For example, birch stood out from the other pollen, as it increased production of IL-8, GM-CSF, IL-1*β*, and TNF-*α* the most. When comparing the properties of the studied pollen, timothy and ragweed seem to have the greatest impact on impairing cytokine-mediated defense against pathogen-like exposures. In previous studies, specifically grass pollen has been shown to activate the release of a variety of chemokines and cytokines in experimental epithelial cell models [17], as well as in human samples [28–30].

Also, other factors than endogenous properties of pollen may contribute to their immunomodulatory potency. For example, pollen may contain microbial components [31–33]. In the study by Obersteiner et al. [31], allergenicity of birch was correlated with the high bacterial diversity on pollen. Manirajan et al. [33] noticed that high allergenic plant pollen (e.g., birch) had a higher amount of endotoxins compared to low allergenic plant pollen. Endotoxins, also called as LPS, are the component of the outer membrane of Gram-negative bacteria and recognized by TLR4 on innate immune cells. Interestingly, it has been observed that the TLR4 signaling pathway may also play a major role in the recognition of pollen [34, 35]. It is possible that cells are already exhausted due to pollen exposure and, thus, show weaker responses to TLR4 stimulation compared to TLR7/8. Altogether, coexposure with TLR7/8 and pollen affected cellular functions the most and this was not dependent on the order in which cells were exposed to these agents.

### 4.3. Strengths and Limitations of the Study and Future Perspectives

To our knowledge, this is the first study assessing how prior pollen exposure affects airway's reactions against viral or bacterial challenge *in vitro*, as well as how prior viral ligand exposure modulates responses against pollen. The strength of our study is that we used pollen from three different plant types that comprehensively represent different allergenic species. Toxicological end points and several immunological mediators were analyzed from cells exposed to three different doses of pollen. We also used three types of viral and bacterial ligands to mimic the effect of different types of pathogens. Some limitations should be considered in future studies. First, similar research setups should be performed using primary cells (e.g., human airway epithelial cells and PBMCs) and *in vivo* models to further disentangle mechanisms of synergistic acts of pollen and pathogens, as well as the importance of cell-to-cell signaling. Second, our study was conducted using pathogen-like ligands, and, thus, further research using respiratory viruses is needed to confirm these interactions and their impact on respiratory health. Although viral ligands have been widely used in many studies, they do not fully express the properties of genuine pathogens, such as their ability to invade and multiply in cells and activate combinations of different TLR pathways. In future studies, other antiviral pathways should also be considered, e.g., dsRNA response, type I IFNs, ISGs, protein synthesis inhibition, and MHC I downregulation. Also, the significance of various receptors operating during pollen and pathogen exposure should be investigated. Last, we acknowledge that the concentrations of pollen used in our study may not be comparable to those typically encountered in nature and that the effects observed *in vitro* may not fully translate to real-life situations. Our study provides a starting point for further investigation into the effects of different pollen types on airway immunity and serves as a foundation for future studies to investigate these effects at more physiologically relevant concentrations.

## 5. Conclusion

This study offers insights into the complex interactions among pollen, respiratory pathogens, and airway cells, providing a better understanding of their influence on immune responses. Our study shows that pollen exposure has the potential to attenuate immune reactions against bacterial- or viral-like challenges by modulating cytokine production. Conversely, TLR7/8-mediated viral challenges, resembling single-stranded RNA viruses like SARS-CoV-2, can induce overly robust immune responses against pollen. Both mechanisms may accelerate and complicate infections during the pollen season. Further research is essential to fully comprehend these interactions and their implications for respiratory health.

## Data Availability

The data that support the findings of this study are available from the corresponding author upon reasonable request

## Abbreviations

A549: Human adenocarcinomic alveolar epithelial cell line
DCF: 2′,7′-dichlorofluorescein
MTT: 3-[4,5-dimethylthiazole-2-yl]-2,5-diphenyltetrazolium bromide
LPS: Lipopolysaccharide
ORN: Synthetic single-stranded RNA oligoribonucleotide
PBS: Phosphate-buffered saline
PI: Propidium iodide
POLY (I:C): Polyinosinic-polycytidylic acid
RNase: Bovine pancreatic ribonuclease
ROS: Reactive oxygen species
THP-1: Human monocytic leukemia cell line
TLR: Toll-like receptor.

## Supplementary Materials

Supplementary Materials Table S1: distributions and detection limits (DL) of studied cytokines in setup 1. Table S2: distributions and detection limits (DL) of studied cytokines in setup 2. Table S3: results of oxidative stress, cellular metabolic activity (CMA), and viability when cocultured cells were first exposed to three doses (0.625, 2.5, 10 mg/ml) of four different pollens (24 hr) and subsequently to Toll-like receptor ligands (24 hr) (setup 1). Table S4: results of oxidative stress, cellular metabolic activity (CMA), and viability results when cocultured cells were first exposed to Toll-like receptor ligands (24 hr) and subsequently three doses (0.625, 2.5, 10 mg/ml) of four different pollens (24 hr) (setup 2). Figure S1: chemokines and growth factor levels when cocultured cells were first exposed to three doses (0.625, 2.5, 10 mg/ml) of four different pollens (24 hr) and subsequently to Toll-like receptor ligand 4 (24 hr) (setup 1) Figure S2: pro- and anti-inflammatory cytokines levels when cocultured cells were first exposed to three doses (0.625, 2.5, 10 mg/ml) of four different pollens (24 hr) and subsequently to Toll-like receptor ligand 4 (24 hr) (setup 1). Figure S3: chemokines and growth factor levels when cocultured cells were first exposed to three doses (0.625, 2.5, 10 mg/ml) of four different pollens (24 hr) and subsequently to Toll-like receptor ligand 3 (24 hr) (setup 1). Figure S4: pro- and anti-inflammatory cytokines levels when cocultured cells were first exposed to three doses (0.625, 2.5, 10 mg/ml) of four different pollens (24 hr) and subsequently to Toll-like receptor ligand 3 (24 hr) (setup 1). Figure S5: chemokines and growth factor levels when cocultured cells were first exposed to Toll-like receptor ligand 4 (24 hr) and subsequently to three doses (0.625, 2.5, 10 mg/ml) of four different pollens (24 hr) (setup 2). Figure S6: pro- and anti-inflammatory cytokines levels when cocultured cells were first exposed to Toll-like receptor ligand 4 (24 hr) and subsequently three doses (0.625, 2.5, 10 mg/ml) of four different pollens (24 hr) (setup 2). Figure S7: chemokines and growth factor levels when cocultured cells were first exposed to Toll-like receptor ligand 3 (24 hr) and subsequently to three doses (0.625, 2.5, 10 mg/ml) of four different pollens (24 hr) (setup 2). Figure S8: pro- and anti-inflammatory cytokines levels when cocultured cells were first exposed to Toll-like receptor ligand 3 (24 hr) and subsequently three doses (0.625, 2.5, 10 mg/ml) of four different pollens (24 hr) (setup 2; *N* ≥ 3).
