# Supplementary material for: Common Pollen Modulate Immune Responses against Viral-Like Challenges in Airway Coculture Model
Source: J Immunol Res. 2023 Nov 6;2023:6639092. doi: 10.1155/2023/6639092 (PMC10643028; doi:10.1155/2023/6639092)
Supplement: Supplementary Materials — Table S1: distributions and detection limits (DL) of studied cytokines in setup 1. Table S2: distributions and detection limits (DL) of studied cytokines in setup 2. Table S3: results of oxidative stress, cellular metabolic activity (CMA), and viability when cocultured cells were first exposed to three doses (0.625, 2.5, 10 mg/ml) of four different pollens (24 hr) and subsequently to Toll-like receptor ligands (24 hr) (setup 1). Table S4: results of oxidative stress, cellular metabolic activity (CMA), and viability results when cocultured cells were first exposed to Toll-like receptor ligands (24 hr) and subsequently three doses (0.625, 2.5, 10 mg/ml) of four different pollens (24 hr) (setup 2). Figure S1: chemokines and growth factor levels when cocultured cells were first exposed to three doses (0.625, 2.5, 10 mg/ml) of four different pollens (24 hr) and subsequently to Toll-like receptor ligand 4 (24 hr) (setup 1) Figure S2: pro- and anti-inflammatory cytokines levels when cocultured cells were first exposed to three doses (0.625, 2.5, 10 mg/ml) of four different pollens (24 hr) and subsequently to Toll-like receptor ligand 4 (24 hr) (setup 1). Figure S3: chemokines and growth factor levels when cocultured cells were first exposed to three doses (0.625, 2.5, 10 mg/ml) of four different pollens (24 hr) and subsequently to Toll-like receptor ligand 3 (24 hr) (setup 1). Figure S4: pro- and anti-inflammatory cytokines levels when cocultured cells were first exposed to three doses (0.625, 2.5, 10 mg/ml) of four different pollens (24 hr) and subsequently to Toll-like receptor ligand 3 (24 hr) (setup 1). Figure S5: chemokines and growth factor levels when cocultured cells were first exposed to Toll-like receptor ligand 4 (24 hr) and subsequently to three doses (0.625, 2.5, 10 mg/ml) of four different pollens (24 hr) (setup 2). Figure S6: pro- and anti-inflammatory cytokines levels when cocultured cells were first exposed to Toll-like receptor ligand 4 (24 hr) and subseq [file 6639092.f1.docx]

**Table S1**. Distributions and detection limits (DL) of studied cytokines in setup 1.

|  |  | | | | | | | | | | | | |
| --- | --- | --- | --- | --- | --- | --- | --- | --- | --- | --- | --- | --- | --- |
|  | | | **Eotaxin** | **GM-CSF** | **IFN-γ** | **IL-10** |  | **IL-1β** | **IL-6** | **MCP-1** | **MDC** | **MIP-1β** | **TNF-α** |
| **N** | | **Valid** | 212 | 212 | 170 | 212 |  | 212 | 212 | 212 | 212 | 212 | 212 |
|  |  | **Missing** | 0 | 0 | 42 | 0 |  | 0 | 0 | 0 | 0 | 0 | 0 |
| **Mean** | | | 144.79 | 52.09 | 22.73 | 13.39 |  | 8.52 | 1930.74 | 5730.65 | 152.55 | 1536.67 | 82.73 |
| **Minimum** | | | 0.00 | 0.98 | 0.00 | 0.94 |  | 0.00 | 13.29 | 50.88 | 3.79 | 4.18 | 0.00 |
| **Maximum** | | | 716.11 | 391.52 | 86.88 | 60.71 |  | 41.24 | 2100.00 | 6290.00 | 548.26 | 1840.00 | 439.35 |
| **DL** | | ***Upper*** | 6210 | 10800 | 28700 | 4280 |  | 4680 | 2100 | 6290 | 18000 | 1840 | 3540 |
|  |  | ***Lower*** | 53.9 | 0.438 | 7.1 | 0.866 |  | 0.514 | 1.42 | 0.465 | 31.8 | 1.01 | 2.49 |

**Table S2**. Distributions and detection limits (DL) of studied cytokines in setup 2.

|  | | | | | | | | | | | |
| --- | --- | --- | --- | --- | --- | --- | --- | --- | --- | --- | --- |
|  | | **Eotaxin** | **GM-CSF** | **IFN-γ** | **IL-10** | **IL-1β** | **IL-6** | **MCP-1** | **MDC** | **MIP-1β** | **TNF-α** |
| **N** | **Valid** | 108 | 188 | 160 | 188 | 188 | 188 | 188 | 188 | 188 | 188 |
|  | **Missing** | 80 | 0 | 28 | 0 | 0 | 0 | 0 | 0 | 0 | 0 |
| **Mean** | | 101.26 | 20.95 | 18.84 | 11.37 | 12.54 | 1309.73 | 5813.90 | 374.03 | 1273.30 | 47.77 |
| **Minimum** | | 0.00 | 0.44 | 0.00 | 0.87 | 0.00 | 70.98 | 1029.70 | 0.00 | 4.18 | 0.00 |
| **Maximum** | | 667.15 | 132.86 | 71.25 | 69.52 | 76.55 | 5693.13 | 6290.00 | 1023.13 | 1840.00 | 221.83 |
| **DL** | ***Upper*** | 6210 | 10800 | 28700 | 4280 | 4680 | 2100 | 6290 | 18000 | 1840 | 3540 |
|  | ***Lower*** | 53.9 | 0.438 | 7.1 | 0.866 | 0.514 | 1.42 | 0.465 | 31.8 | 1.01 | 2.49 |

**Table S3**. Oxidative stress, cellular metabolic activity (CMA), and viability results when cocultured cells were first exposed to three doses (0.625, 2.5, 10 mg/ml) of four different pollens (24 hr), and subsequently to Toll-like receptor ligands (24 hr) (setup 1*, N ≥ 3*). Table shows mean ± SEM. Significance was assumed at p < 0.05.

|  |  | **Oxidative stress**  **(% of control)** | **CMA**  **(% of control)** | **Viability**  **(% of control)** |
| --- | --- | --- | --- | --- |
| **Ligands** | TLR3 | 105.0 ± 3.3 | 101.7 ± 1.6 | 99.8 ± 3.7 |
|  | TLR4 | 105.3 ± 3.1 | 97.9 ± 1.8 | 102.4 ± 4.9 |
|  | TLR7/8 | 104.3 ± 3.3 | 95.5 ± 1.9 | 105.1 ± 3.2 |
| **Alder** | 0.625 mg/ml | 104.3 ± 3.7 | 110.4 ± 2.4 | 96.5 ± 2.8 |
|  | *+TLR3* | 103.1 ± 5.4 | 105.8 ± 3.8 | 110.2 ± 8.6 |
|  | *+TLR4* | 96.7 ± 3.8 | 106.2 ± 2.3 | 90.4 ± 5.4 |
|  | *+TLR7/8* | **119.1 ± 2.1 ^a^** | 104.6 ± 2.5 | 120.9 ± 8.5 |
|  | 2.5 mg/ml | 113.2 ± 3.4 | 109.3 ± 2.6 | 99.0 ± 4.7 |
|  | *+TLR3* | 100.7 ± 3.9 | 99.1 ± 3.7 | 101.7 ± 8.0 |
|  | *+TLR4* | 101.4 ± 4.1 | 103.7 ± 3.6 | 98.5 ± 4.2 |
|  | *+TLR7/8* | **124.7 ± 3.4 ^a^** | 107.3 ± 2.0 | 117.3 ± 6.4 |
|  | 10 mg/ml | 94.2 ± 4.1 | 87.6 ± 3.1 | 90.8 ± 4.1 |
|  | *+TLR3* | **83.4 ± 4.0 ^b^** | 76.8 ± 6.4 | 77.7 ± 9.7 |
|  | *+TLR4* | 92.3 ± 5.6 | 86.2 ± 5.5 | 85.9 ± 7.7 |
|  | *+TLR7/8* | **112.3 ± 3.9 ^a^** | **97.8 ± 2.9 ^a^** | 104.0 ± 9.6 |
| **Birch** | 0.625 mg/ml | 102.9± 2.9 | 108.3 ± 2.8 | 102.0 ± 1.2 |
|  | *+TLR3* | 99.8 ± 3.0 | 107.3 ± 3.4 | 102.7 ± 3.2 |
|  | *+TLR4* | 99.8 ± 6.2 | 100.6 ± 1.6 | 104.0 ± 2.8 |
|  | *+TLR7/8* | **115.7 ± 2.7 ^a^** | 101.8 ± 6.3 | 101.1 ± 1.6 |
|  | 2.5 mg/ml | 120.8 ± 4.0 | 104.0 ± 4.3 | 98.5 ± 2.7 |
|  | *+TLR3* | 106.6 ± 3.5 | 111.2 ± 3.0 | 99.4 ± 2.6 |
|  | *+TLR4* | 106.5 ± 2.7 | 102.3 ± 2.1 | 95.8 ± 2.2 |
|  | *+TLR7/8* | 124.1 ± 5.6 | 112.6 ± 6.6 | 99.8 ± 4.5 |
|  | 10 mg/ml | 114.8 ± 5.0 | 89.4 ± 3.1 | 94.9 ± 3.0 |
|  | *+TLR3* | 111.4 ± 4.1 | **101.7 ± 1.9 ^a^** | 98.1 ± 7.3 |
|  | *+TLR4* | 111.0 ± 4.7 | 95.6 ± 2.1 | 102.0 ± 1.3 |
|  | *+TLR7/8* | 124.3 ± 4.3 | **102.0 ± 4.3 ^a^** | 98.6 ± 6.7 |
| **Timothy** | 0.625 mg/ml | 91.7 ± 3.4 | 101.6 ± 2.3 | 105.6 ± 7.7 |
|  | *+TLR3* | **85.5 ± 3.4 ^b^** | 112.6 ± 5.3 | 109.8 ± 4.5 |
|  | *+TLR4* | 98.1 ± 3.3 | 103.3 ± 2.6 | 114.0 ± 6.0 |
|  | *+TLR7/8* | **92.4 ± 4.6 ^b^** | 101.3 ± 5.7 | 101.3 ± 4.4 |
|  | 2.5 mg/ml | 82.7 ± 3.4 | 87.1 ± 2.2 | 105.3 ± 6.5 |
|  | *+TLR3* | **69.7 ± 3.6 ^b^** | 93.1 ± 2.2 | 111.1 ± 4.5 |
|  | *+TLR4* | **81.2 ± 4.8 ^b^** | 87.0 ± 2.9 | 102.4 ± 7.3 |
|  | *+TLR7/8* | **86.0 ± 4.1 ^b^** | 86.9 ± 3.2 | 101.0 ± 3.5 |
|  | 10 mg/ml | 45.1 ± 2.9 | 53.7 ± 3.1 | 105.3 ± 6.9 |
|  | *+TLR3* | **32.8 ± 1.5 ^a b^** | **49.0 ± 3.1 ^b^** | 111.4 ± 8.8 |
|  | *+TLR4* | **35.6 ± 3.7 ^b^** | **54.3 ± 4.0 ^b^** | 110.8 ± 8.2 |
|  | *+TLR7/8* | **48.63 ± 3.6 ^b^** | **61.4 ± 3.4 ^b^** | 104.2 ± 9.1 |
| **Ragweed** | 0.625 mg/ml | 121.9 ± 3.9 | 102.5 ± 3.5 | 104.6 ± 4.3 |
|  | *+TLR3* | 112.5 ± 4.5 | 104.8 ± 2.3 | 107.0 ± 5.9 |
|  | *+TLR4* | **105.0 ± 6.5 ^a^** | 102.5 ± 3.9 | 109.8 ± 9.7 |
|  | *+TLR7/8* | 131.7 ± 5.3 | **91.5 ± 2.1 ^a^** | 107.1 ± 5.6 |
|  | 2.5 mg/ml | 125.6 ± 5.3 | 84.9 ± 2.4 | **106.7 ± 7.1 ^b^** |
|  | *+TLR3* | 121.30 ± 8.6 | 89.0 ± 2.4 | 85.5 ± 6.9 |
|  | *+TLR4* | 132.8 ± 10.8 | 89.5 ± 2.1 | 104.3 ± 7.5 |
|  | *+TLR7/8* | **173.2 ± 7.4 ^a^** | 84.3 ± 2.6 | 101.3 ± 3.6 |
|  | 10 mg/ml | 54.8 ± 4.2 | 41.3 ± 2.1 | 78.5 ± 7.9 |
|  | *+TLR3* | **55.11 ± 4.5  ^b^** | **45.3 2.3 ^b^** | **70.2 ± 6.2  ^b^** |
|  | *+TLR4* | **50.1 ± 5.5  ^b^** | **45.5 ± 2.6 ^b^** | **74.1 ± 12.7 ^b^** |
|  | *+TLR7/8* | **100.9 ± 6.5 ^a^** | **42.4 ± 1.7  ^b^** | 84.5 ± 7.1 |

**Table S4**. Oxidative stress, cellular metabolic activity (CMA), and viability results when cocultured cells were first exposed to Toll-like receptor ligands (24 hr), and subsequently three doses (0.625, 2.5, 10 mg/ml) of four different pollen (24 hr) (setup 2, *N ≥ 3)*. Table shows mean ± SEM. Significance was assumed at p < 0.05.

|  | |  | | | | |
| --- | --- | --- | --- | --- | --- | --- |
|  |  | | **Oxidative stress**  **(% of control)** | **CMA**  **(% of control)** | **Viability**  **(% of control)** |  |
| **Ligands** | TLR3 | | 108.2 ± 1.9 | 105.1 ± 4.0 | 103.9 ± 3.0 |  |
|  | TLR4 | | 106.9 ± 4.5 | 108.6 ± 3.8 | 107.8 ± 6.1 |  |
|  | TLR7/8 | | 110.9 ± 4.0 | 105.9 ± 2.6 | 110.1 ± 5.8 |  |
| **Alder** | 0,625 mg/ml | | 97.7 ± 2.2 | 98.7 ± 0.7 | 100.8 ± 3.2 |  |
|  | *+TLR3* | | 104.6 ± 5.8 | 98.2 ± 4.0 | 105.6 ± 5.6 |  |
|  | *+TLR4* | | 105.3 ± 7.2 | 97.0 ± 4.2 | 111.1 ± 6.2 |  |
|  | *+TLR7/8* | | 115.0 ± 4.9 | 105.9 ± 3.7 | 114.0 ± 6.5 |  |
|  | 2,5 mg/ml | | 109.6 ± 5.8 | 104.0 ± 2.2 | 101.8 ± 2.0 |  |
|  | *+TLR3* | | 102.7 ± 2.5 | 98.2 ± 2.5 | 103.7 ± 6.7 |  |
|  | *+TLR4* | | 104.7 ± 3.0 | 96.8 ± 1.4 | 107.0 ± 2.7 |  |
|  | *+TLR7/8* | | 117.0 ± 3.3 | 96.1 ± 4.3 | 114.9 ± 7.3 |  |
|  | 10 mg/ml | | 104.2 ± 3.6 | 95.4 ± 2.8 | 102.5 ± 4.5 |  |
|  | *+TLR3* | | 102.8 ± 3.8 | 100.7 ± 2.3 | 99.9 ± 1.8 |  |
|  | *+TLR4* | | 112.5 ± 3.1 | 100.2 ± 3.9 | 102.5 ± 1.7 |  |
|  | *+TLR7/8* | | **131.8 ± 4.9 ^b^** | 99.7 ± 3.7 | 114.1 ± 6.9 |  |
| **Birch** | 0,625 mg/ml | | 116.2 ± 12.0 | 110.9 ± 2.3 | 92.9 ± 11.6 |  |
|  | *+TLR3* | | 91.8 ± 6.7 | 129.6 ± 8.5 | 118.5 ± 17.0 |  |
|  | *+TLR4* | | 128.4 ± 11.7 | 111.3 ± 4.6 | 113.0 ± 11.1 |  |
|  | *+TLR7/8* | | 116.0 ± 7.1 | 123.5 ± 2.6 | 97.8 ± 14.7 |  |
|  | 2,5 mg/ml | | 120.8 ± 9.1 | 117.1 ± 7.1 | 102.6 ± 6.5 |  |
|  | *+TLR3* | | 90.2 ± 8.1 | **137.5 ± 10.4 ^b^** | 111.4 ± 21.4 |  |
|  | *+TLR4* | | **130.2 ± 9.3 ^b^** | 117.5 ± 5.7 | 111.5 ± 10.3 |  |
|  | *+TLR7/8* | | 116.8 ± 6.1 | 115.7 ± 5.0 | 101.9 ± 9.6 |  |
|  | 10 mg/ml | | 133.7 ± 6.9 | 111.4 ± 3.6 | 103.5 ± 9.8 |  |
|  | *+TLR3* | | 105.6 ± 15.5 | 142.4 ± 16.5 | 93.9 ± 10.6 |  |
|  | *+TLR4* | | 105.8 ± 10.0 | 119.0 ± 6.4 | 104.2 ± 12.1 |  |
|  | *+TLR7/8* | | **172.7 ± 10.6 ^b^** | 120.3 ± 6.1 | 101.2 ± 9.6 |  |
| **Timothy** | 0,625 mg/ml | | 97.9 ± 4.2 | 102.2 ± 2.9 | 109.4 ± 6.7 |  |
|  | *+TLR3* | | 106.3 ± 7.1 | 105.8 ± 7.8 | 102.7 ± 7.1 |  |
|  | *+TLR4* | | 105.2 ± 4.0 | 103.7 ± 3.1 | 117.1 ± 5.9 |  |
|  | *+TLR7/8* | | 110.1 ± 9.5 | 97.0 ± 2.5 | 113.3 ± 10.3 |  |
|  | 2,5 mg/ml | | 104.6 ± 3.6 | 109.6 ± 4.1 | 110.3 ± 4.1 |  |
|  | *+TLR3* | | 103.8 ± 6.9 | 99.3 ± 6.9 | 97.7 ± 3.5 |  |
|  | *+TLR4* | | 107.4 ± 6.1 | 108.3 ± 6.9 | 116.9 ± 6.3 |  |
|  | *+TLR7/8* | | 105.8 ± 4.7 | **91.8 ± 2.5 ^b^** | 112.0 ± 10.7 |  |
|  | 10 mg/ml | | 100.2 ± 3.7 | 105.7 ± 2.7 | 110.1 ± 5.7 |  |
|  | *TLR3* | | 108.8 ± 12.0 | 105.4 ± 6.1 | 107.5 ± 8.2 |  |
|  | *TLR4* | | 103.2 ± 5.5 | 108.9 ± 5.7 | 112.5 ± 6.5 |  |
|  | *TLR7/8* | | 101.7 ± 4.4 | 101.6 ± 4.2 | 111.9 ± 9.6 |  |
| **Ragweed** | 0,625 mg/ml | | 122.3 ± 8.9 | 108.0 ± 2.8 | 101.9 ± 11.8 |  |
|  | *+TLR3* | | 116.9 ± 5.22 | 101.5 ± 3.3 | 104.9 ± 13.3 |  |
|  | *+TLR4* | | 111.2 ± 9.9 | **91.4 ± 3.8 ^ab^** | 101.8 ± 17.3 |  |
|  | *+TLR7/8* | | 119.6 ± 8.7 | **93.0 ± 3.1 ^ab^** | 105.1 ± 8.5 |  |
|  | 2,5 mg/ml | | 127.3 ± 9.0 | 98.5 ± 5.2 | 99.7 ± 11.8 |  |
|  | *+TLR3* | | 132.0 ± 6.5 | 93.4 ± 2.5 | 103.6 ± 13.0 |  |
|  | *+TLR4* | | **139.3 ± 10 ^b^** | **95.9 ± 0.7 ^b^** | 109.6 ± 11.2 |  |
|  | *+TLR7/8* | | 126.8 ± 9.9 | **88.0 ± 5.7 ^b^** | 108.4 ± 21.1 |  |
|  | 10 mg/ml | | 132.1 ± 12.8 | 80.1 ± 3.3 | 96.2 ± 11.3 |  |
|  | *+TLR3* | | 135.2 ± 10.7 | 92.0 ± 3.4 | 105.0 ± 11.9 |  |
|  | *+TLR4* | | **147.5 ± 17.3 ^b^** | **83.2 ± 2.6 ^b^** | 107.8 ± 11.7 |  |
|  | *+TLR7/8* | | **142.4 ± 5.8 ^b^** | **80.3 ± 2.0 ^b^** | 93.3 ± 8.5 |  |

***Figure S1. Chemokines and growth factor*** *levels when cocultured cells were first exposed to three doses (0.625. 2.5. 10 mg/ml) of four different pollens (24 hr) and subsequently to Toll-like receptor ligand 4 (24 hr) (setup 1. N ≥ 3). Figure shows mean ± SEM. a indicates significance from pollen. b indicates significance from TLR4.*

***Figure S2. Pro- and anti-inflammatory*** *cytokines levels* *when cocultured cells were first exposed to three doses (0.625. 2.5. 10 mg/ml) of four different pollens (24 h) and subsequently to Toll-like receptor ligand 4 (24 h) (setup 1. N ≥ 3). Figure shows mean ± SEM. a indicates significance from pollen. b indicates significance from TLR4.*

***Figure S3.*** ***Chemokines and growth factor*** *levels when co-cultured cells were first exposed to three doses (0.625. 2.5. 10 mg/ml) of four different pollens (24 hr). and subsequently to Toll-like receptor ligand 3 (24 hr) (setup 1. N ≥ 3). Figure shows mean ± SEM. a indicates significance from pollen. b indicates significance from TLR3.*

***Figure S4.*** ***Pro- and anti-inflammatory*** *cytokines levels when co-cultured cells were first exposed to three doses (0.625. 2.5. 10 mg/ml) of four different pollens (24 hr) and subsequently to Toll-like receptor ligand 3 (24 hr) (setup 1. N ≥ 3). Figure shows mean ± SEM. a indicates significance from pollen. b indicates significance from TLR3.*

***Figure S5.*** ***Chemokines and growth factor*** *levels when co-cultured cells were first exposed to Toll-like receptor ligand 4 (24 hr). and subsequently to three doses (0.625. 2.5. 10 mg/ml) of four different pollens (24 hr) (setup 2. N ≥ 3). Figure shows mean ± SEM. a indicates significance from pollen. b indicates significance from TLR4.*

***Figure S6.*** ***Pro- and anti-inflammatory cytokines*** *levels when co-cultured cells were first exposed to Toll-like receptor ligand 4 (24 hr) and subsequently three doses (0.625. 2.5. 10 mg/ml) of four different pollens (24 hr) (setup 2. N ≥ 3). Figure shows mean ± SEM. a indicates significance from pollen. b indicates significance from TLR4.*

***Figure S7.*** ***Chemokines and growth factor*** *levels when co-cultured cells were first exposed to Toll-like receptor ligand 3 (24 hr) and subsequently to three doses (0.625. 2.5. 10 mg/ml) of four different pollens (24 hr) (setup 2. N ≥ 3). Figure shows mean ± SEM. a indicates significance from pollen. b indicates significance from TLR3.*

***Figure S8.*** ***Pro- and anti-inflammatory cytokines*** *levels when co-cultured cells were first exposed to Toll-like receptor ligand 3 (24 hr) and subsequently three doses (0.625. 2.5. 10 mg/ml) of four different pollens to for another (24 hr) (setup 2. N ≥ 3). Figure shows mean ± SEM. a indicates significance from pollen. b indicates significance from TLR3.*
